# Kidney biopsy findings in two patients with TAFRO syndrome: case presentations and review of the literature

**DOI:** 10.1186/s12882-020-02119-7

**Published:** 2020-11-23

**Authors:** Qianyun Zhou, Yuanyuan Zhang, Guangping Zhou, Jihong Zhu

**Affiliations:** 1 Intensive Care Unit Peking University Shougang Hospital, Beijing, China; 2 Department of Emergency, Peking University People’s Hospital, Beijing, China

**Keywords:** TAFRO syndrome, Castleman disease, Renal insufficiency, Lymph node, Kidney, Biopsy

## Abstract

**Background:**

TAFRO syndrome is a clinical subtype of idiopathic multicentric Castleman disease (iMCD) that is characterized by thrombocytopenia, anasarca, fever, reticulin myelofibrosis (or renal dysfunction), and organomegaly. TAFRO syndrome has only recently been described, and many clinicians are unaware of this disease, leading to delays in diagnosis and treatment. We present two patients with TAFRO syndrome in whom renal biopsies were performed.

**Case presentation:**

Both patients had subacute onset and exhibited renal insufficiency, edema, anemia, thrombocytopenia, polyserositis and lymphadenopathy over the disease course. However, there were many differences in their clinical manifestations. Case 1 was a 30-year-old woman admitted due to intermittent vaginal bleeding for 3 weeks. Laboratory tests on admission showed severe renal insufficiency (creatinine: 624 μmol/L), severe anemia (Hb: 41 g/L), and moderate thrombocytopenia (61 × 10^9^/L). Case 2 was a 42-year-old man. Acute epigastric pain was his initial complaint, and computed tomography (CT) revealed retroperitoneal exudation around the pancreas. He was diagnosed with acute pancreatitis, and after treatment with a proton pump inhibitor (PPI) and somatostatin, his abdominal pain still recurred. During treatment, renal failure gradually increased, with oliguria, fever, anemia, thrombocytopenia, edema and massive ascites. Lymph node histologies were consistent with the hyaline-vascular (HV) type and mixed type, respectively, and renal histopathologies were consistent with thrombotic microangiopathy (TMA)-like renal lesions and membranoproliferative glomerulonephritis (MPGN), respectively. Their general conditions improved after glucocorticoid therapy, but their renal functions did not recover completely. On the basis of glucocorticoids, second-line treatments with tocilizumab and rituximab, respectively, were applied.

**Conclusions:**

The diagnosis of TAFRO syndrome is based mainly on clinical manifestations and lymph node biopsies. A reliable early diagnosis and appropriate rapid treatment are essential to improve patient outcomes. Clinicians should deepen their understanding of this disease and similar conditions. Once the disease is suspected, lymph node biopsies should be performed as soon as possible. In addition, renal biopsies should be actively performed in patients with renal involvement.

## Background

Castleman disease (CD) was first reported in 1954 by Prof. Castleman and was initially described as a lymphoproliferative disorder with a peculiar histological pattern observed in a large mediastinal mass [1]. In the following decades, “variants” were described according to several dichotomies: the hyaline vascular (HV) type versus the plasmacytic (PC) type [2], unicentric CD (UCD) versus multicentric CD (MCD) [3, 4], and human immunodeficiency virus (HIV)/herpesvirus (HHV)-8 positive versus HIV/HHV-8 negative [5, 6]. Currently, HIV/HHV-8-negative multicentric CD is known as idiopathic multicentric CD (iMCD) [7].

In 2010, Takai et al. [8] described three patients who shared several abnormal laboratory test results and clinical features, including thrombocytopenia, anasarca (edema, pleural effusion and ascites), fever, reticulin myelofibrosis and organomegaly (hepatosplenomegaly and lymphadenopathy), and this new clinical entity was referred to as TAFRO syndrome. Of note, one of these three patients underwent lymph node biopsy demonstrating hyaline-vascular-like changes consistent with MCD. Since the initial description by Takai et al., similar cases have been reported [9–14]. To date, most studies and case reports on TAFRO syndrome have been performed by Japanese scholars, and the most commonly cited diagnostic criteria for TAFRO syndrome were proposed by Iwaki et al. in 2016 [15]. In their paper, they recommended further subclassification of iMCD into TAFRO-iMCD and iMCD-NOS (not otherwise specified) and proposed the following histopathological features characteristic of TAFRO-iMCD: lymph nodes with atrophic germinal centers and enlarged endothelial cell nuclei, proliferation of endothelial venules with enlarged nuclei in the interfollicular zone, and small numbers of mature plasma cells [10]. According to their diagnostic criteria, two histopathological criteria (compatible with the pathological findings of lymph nodes as TAFRO-iMCD and LANA-1 and HHV-8 negative) and three major criteria (presents with 3 of 5 TAFRO syndrome characteristics, absence of hypergammaglobulinemia, and small volume lymphadenopathy) must be fulfilled; moreover, 1 or more of the minor criteria (hyper/normoplasia of megakaryocytes in the bone marrow and high levels of serum alkaline phosphatase (ALP) without markedly elevated levels of serum transaminase) needs to be satisfied. The same year, Masaki et al. took a slightly different approach to the diagnosis of TAFRO syndrome [16]. According to their criteria, Castleman’s disease-like features on lymph node biopsy are not necessary for diagnosis, but one of the four minor criteria (the other three criteria are reticulin myelofibrosis and/or an increased number of megakaryocytes in the bone marrow, mild organomegaly, and progressive renal insufficiency) must be satisfied. A diagnosis of TAFRO syndrome requires at least two of the four minor categories in addition to three major categories (anasarca, thrombocytopenia, and systemic inflammation). Therefore, in patients whose lymph node biopsy is difficult to perform due to anasarca, bleeding tendency, and a small target lymph node, the diagnosis can be made based on their clinical presentation. The differential diagnosis of TAFRO syndrome involves diagnostic criteria, including rheumatologic diseases such as SLE; infections such as acute Epstein-Barr virus; and neoplastic diseases such as lymphoma, POEMS (i.e., polyneuropathy, organomegaly, endocrinopathy, monoclonal protein, and skin changes) syndrome, and other cancers.

As a newly recognized disease entity, TAFRO syndrome is unfamiliar to many clinicians, especially those other than hematologists. However, early and reliable diagnoses and early treatments with appropriate agents are essential for favorable outcomes. To enrich our understanding of the clinical features and histopathological findings of this disease entity, we describe two patients diagnosed with TAFRO syndrome who underwent renal biopsy and a review of the literature.

## Case presentations

### Case 1

The patient was a 30-year-old woman who was admitted to our institution due to a 3-week history of intermittent vaginal bleeding. She had a medical history of hypertension for 2 years and regular menses (gravida 0 para 0). On admission, her blood pressure was 149/86 mmHg, her heart rate was 125 beats/min, and her temperature was 38.1 °C. A physical examination revealed moderate pitting edema in both lower limbs. A routine blood test performed in a nearby hospital 4 days before admission showed leukocytosis, severe anemia and thrombocytopenia (Table 1). Laboratory investigations in our hospital revealed the following: C-reactive protein (CRP): > 200 mg/L; hyperplastic anemia (41 g/L); blood urea nitrogen: 39.21 mmol/L; creatinine: 624 μmol/L (7.06 mg/dL); hypoalbuminemia (25.4 g/L); alkaline phosphatase (ALP): 332 U/L; gamma glutamyltransferase: 501 U/L; uric acid: 968 μmol/L; transaminase: normal; fibrinogen: 750 mg/dL; activated partial thromboplastin time (APTT): 70.3 s; plasma correcting test: 37.3 s of immediate APTT and 48.5 s of incubated APTT; autoantibodies [antinuclear antibody, rheumatoid factor, anti-double-stranded DNA antibody, anticardiolipin antibody, anti-neutrophil cytoplasmic antibody, anti-glomerular basement membrane antibody, etc.]: negative; immunoglobulin (IgA, IgG, and IgM): normal; β2 microglobulin: > 8 mg/L (1.00–3.00); M protein: negative; complement 3 and 4: decreased; direct antiglobulin test (DAT): 3+; and procalcitonin: 43.63 ng/ml. Broken red blood cells were not observed on the blood smear. The blood culture, G/GM test and viral detection (Epstein-Barr virus, cytomegalovirus, parvovirus B-19, epidemic hemorrhagic fever, HIV, etc.) test were negative. The urinary sediment examination showed the following: urinary protein: 1+; occult blood: 2+, and hematuria: 56/μl. The 24-h urinary protein level was not checked because of oliguria. Ultrasound scans of the urinary and gynecological systems were normal. Plain computed tomography revealed a small amount of polyserositis and lymphadenopathy in multiple nodes but no evidence of malignant solid tumors or signs of infection. Bone marrow aspiration showed bone marrow hyperplasia, and immunohistochemistry was normal. ^18^FDG-positron emission tomography/computed tomography revealed that glucose analog 2-(^18^F)fluoro-2-deoxy-D-glucose activity was increased in the mediastinal, paraaortic, bilateral neck, bilateral axillary, and bilateral inguinal lymph nodes (SUVmax: 5.2). The largest node measured approximately 2.7 cmx1.2 cm, and hepatosplenomegaly with increased FDG activity was observed in the spleen (SUVmax: 5.2).

**Table 1:** Laboratory data of Case1 and Case 2

Laboratory data	Case 1		Case 2		Normal value
	The first data	The worst data	The first data	The worst data	
WBC(×109/L)	26.2		5.81		3.5–9.5
Hemoglobin (g/L)	52	41	158	67	115–150
Platelets (×109/L)	60	5	205	10	125–350
ALP(U/L)	332		89	202	
Albumin (g/L)	25.4		46.3	23.2	40.0–55.0
Creatinine (μmol/L)	624		88.9	275	45–84
PT(s)	14		13.1		9.4–12.5
Fibrinogen (mg/dL)	750		629		200–400
APTT(s)	70.3		37.4		25.4–38.4
D-dimer (ng/mL)	1096		5370		0–243
CRP (mg/L)	> 200		137		0–10
DAT	3+				Negative
Immunoglobulin	Normal		Normal		Normal
Monoclonal protein	Negative		Negative		Negative
Peripheral broken red blood cells	Negative				Negative
sCD25+ (pg/ml)			10615		< 6400
NK cell activity (%)			14.09		> 15.11
IgG subclass	Normal		Normal		Normal
HHV-8	Negative		Negative		Negative
IL-6 (pg/ml)	9.5		Increased		0–7
Ascitic IL-6 (pg/ml)			1022		0–7
VEGF (pg/ml)	231.47		> 800		0–142

*DAT* direct antiglobulin test, *HHV-8* herpesvirus 8, *-IL-6* interleukin 6, *VEGF* vascular endothelial growth factor

**Fig. 1:**
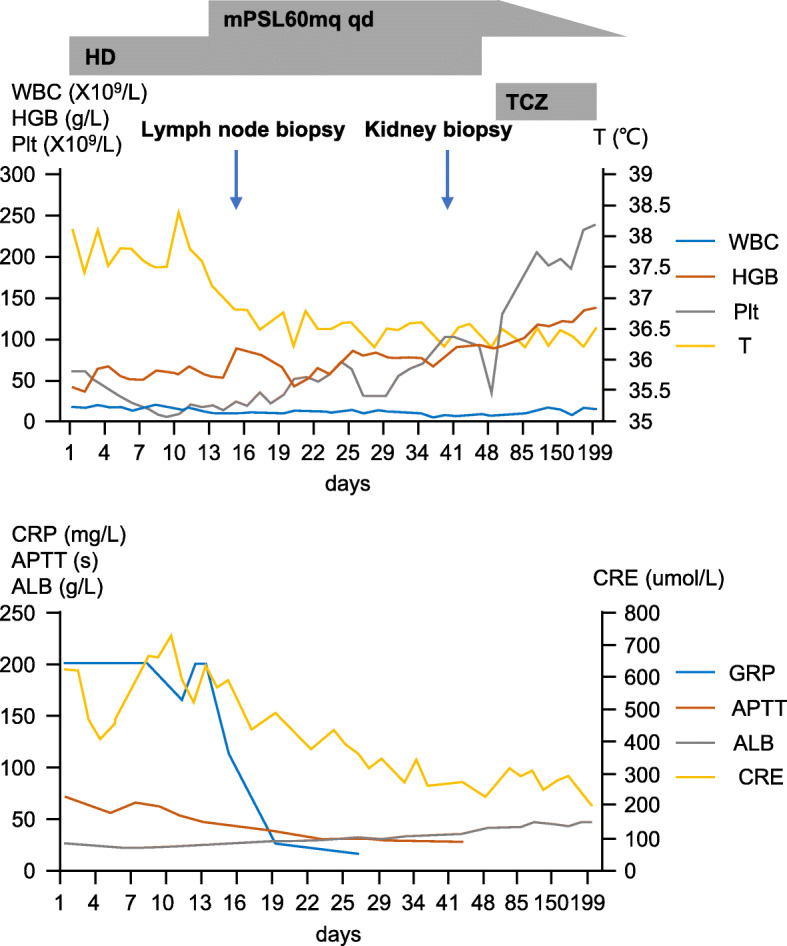
Case 1. Changes in the main clinical indexes over time. WBC: white blood cell. HGB: hemoglobin. Plt: platelet. T: temperature. CRP: C-reactive protein. APTT: activated partial thromboplastin time. ALB: albumin. CRE: creatinine

On admission, the patient was treated with the following: 1) oral Diane-35 (ethinylestradiol cyproterone), 1 tablet q 8 h in the first week and 1 tablet qd in weeks 2–4; 2) regular hemodialysis, three times a week; 3) intravenous anti-infection with meropenem (0.5 q 12 h on day 2) and moxifloxacin (0.4 qd on day 2 and replaced with linezolid on day 7); and 4) blood transfusion as needed (RBC: 800 ml; plasma: 200 ml (total)). After the above treatment, her condition did not improve, with an intractable decrease in hemoglobin and platelets, and her CRP level continued to be above 200 mg/L (Fig. 1).

**Fig. 2:**
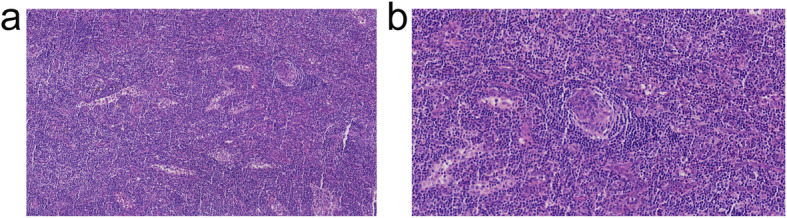
Light microgram showing the histological findings of the left cervical lymph node of case 1. **a** × 200 Hematoxylin-eosin (HE) stain. **b** × 400 Hematoxylin-eosin (HE) stain

The data described above (no evidence of a solid tumor, negative etiologic test and ineffective antibiotic therapy, and no evidence of hemolysis that favored TTP and HUS) indicated a differential diagnosis. Considering her positive DAT and APTT correction test, the latter indicated the presence of a coagulation factor inhibitor in the blood; therefore, we concluded that the autoimmune mechanism was involved in her pathogenesis. Therefore, intravenous methylprednisolone (60 mg qd) was given on day 13. Fortunately, her temperature decreased to normal on day 14, and improvements in laboratory tests were found (i.e., CRP and APTT decreased to normal levels); however, anemia and thrombocytopenia improved relatively slowly. Though severe thrombocytopenia (14 × 10^9^/L) was observed on day 14, lymph node biopsy was performed on the basis of platelet transfusion on day 15. The results showed atrophic germinal centers and obvious proliferation of endothelial vessels in the T-zone. The histological findings were in accordance with the hyaline-vascular (HV) type of CD (Fig. 2). During this period, glucocorticoid therapy alone resulted in a progressive improvement in anemia and thrombocytopenia. Further laboratory tests showed mild to moderate increases in interleukin 6 (IL-6) and vascular endothelial growth factor (VEGF) (9.5 pg/ml and 231.47 pg/ml, respectively), normal levels of IgG4 and negative HHV-8 PCR. Based on these findings, her clinicopathological findings met both Iwaki’s and Masaki’s diagnostic criteria for TAFRO syndrome.

**Fig. 3:**
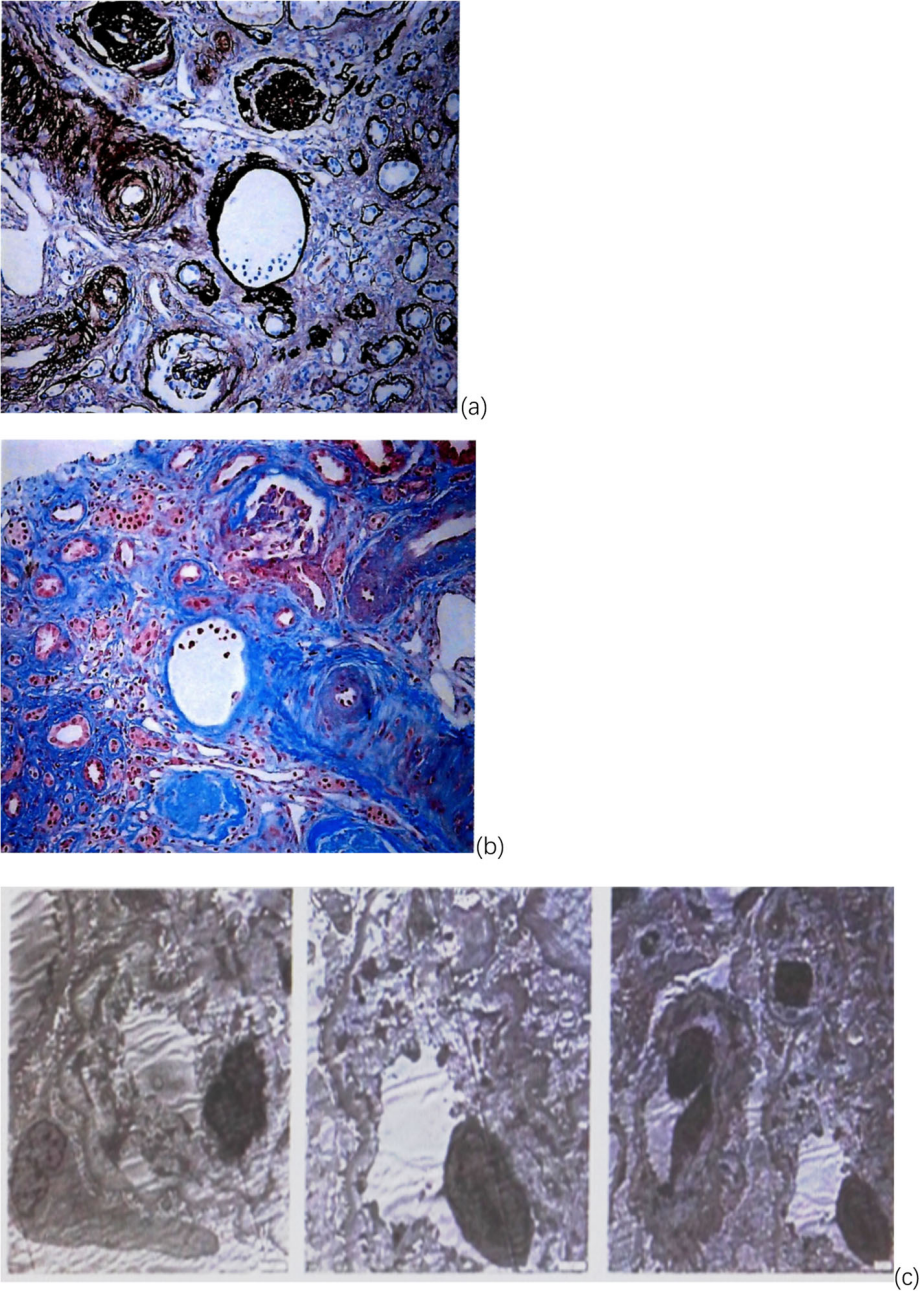
Renal histopathological findings of case 1. **a** Light microgram × 200 periodic acid silver-methenamine (PASM) stain. **b** Light microgram × 200 Masson stain. **c** Electron micrograph × 5000

Due to persistent thrombocytopenia and concerns about the risk of bleeding, renal biopsy was not performed until day 40. Histopathology showed 17 glomeruli, 7 of which were ischemic sclerosis. The mesangial cells and matrixes of the remainder of the glomeruli showed slightly diffuse proliferation and local aggravated endothelial cell proliferation. The basement membrane was thickened heterogeneously, and some of the glomerular basement membrane was ischemic and shrunken. Vacuole and granular degeneration was observed in renal tubular epithelial cells, with expansion of the lumen and diffuse atrophy. The arteriole had an “onion skin” appearance, with a thickened wall and narrowed lumen. Immunofluorescence staining showed that immunoglobulin (IgM++) was deposited in clumps or granules along the mesangial area and capillary wall. Electron microscopy did not reveal any electron-dense deposits but showed widening of the loose layer in the glomerular basement membrane with the accumulation of cell debris. Based on these findings, the patient was diagnosed with thrombotic microangiopathy (TMA)-like renal lesions with subacute renal tubular interstitial nephropathy (Fig. 3). Her urine output increased gradually (approximately 1000 ml/d), and the dialysis interval was prolonged. However, her platelet level decreased again on day 48. On day 50, she accepted intravenous tocilizumab (TCZ, anti-IL-6 receptor monoclonal antibody, from Roche) (8 mg/kg qw in the first month and then once a month). She stopped dialysis on day 65. During the 13-month follow-up, her laboratory tests, including routine blood tests, ALP, CRP and APTT, were all normal, but her creatine level did not decrease to normal levels (249 μmol/L 6 months after admission).

### Case 2

**Fig. 4:**
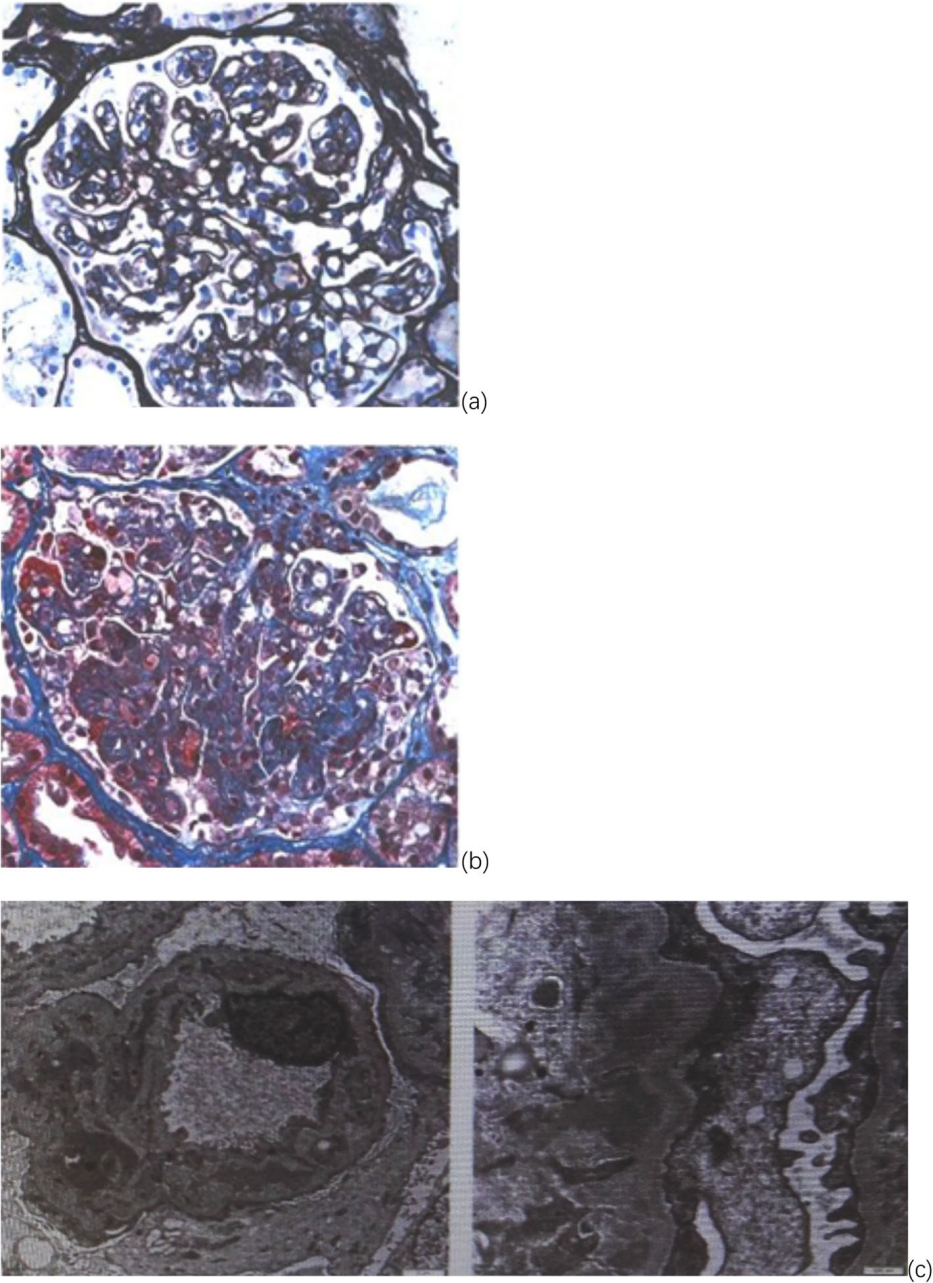
Renal histopathological findings of case 2. **a** Light microgram × 400 periodic acid silver-methenamine (PASM) stain. **b** Light microgram × 200 Masson stain. **c** Electron micrograph × 5000

**Fig. 5:**
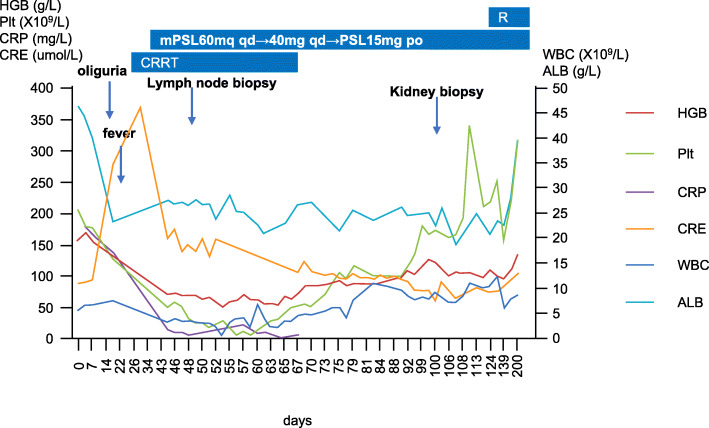
Changes in clinical indicators over time in case 2. WBC: white blood cell. HGB: hemoglobin. Plt: platelet. T: temperature. CRP: C-reactive protein. APTT: activated partial thromboplastin time. ALB: albumin. CRE: creatinine

This patient was a 42-year-old man who had been admitted to several hospitals before being transferred to our hospital for further care. Acute epigastric pain was his initial complaint, and laboratory findings from nearby hospitals, which included routine blood tests, ALP, amylase, transaminase and creatinine, were normal (Table 1). Computed tomography revealed a small amount of retroperitoneal exudation around the body of the pancreas. A combination of a proton pump inhibitor, anisodamine and tramadol was administered, but his symptoms recurred. Six days after onset, he visited another hospital for further care. His alanine transaminase and γ-GGT levels were elevated (118 U/L and 146 U/L, respectively), but his amylase and lipase levels were normal. Because peripancreatic exudation was observed on the CT scan, he was admitted with the diagnosis of acute pancreatitis. After admission, his creatinine level gradually increased (6 days after onset: 89.9 μmol/l; 7 days after onset: 93.9 μmol/l), and the urinary sediment examination showed the following: urinary protein: 2+; and hematuria: 0–2/μL. Nine days after onset, he complained of abdominal colic pain and had diarrhea, and new ascites was found on the plain abdominal CT scan. Eleven days after onset, enhanced CT revealed that the amount of ascites had increased. Fourteen days after onset, he began to have progressive oliguria. Twenty-one days after onset, his illness was aggravated, with a urine output of approximately 200 ml/d, a CRP level of 137 mg/L, a creatinine level of 275 μmol/l, an albumin level of 23.2 g/L and an ALP level of 202 U/L. Therefore, he was transferred to a third hospital that specializes in kidney disease. He began treatment with intermittent bedside hemodiafiltration because of water overload and symptoms of heart failure 24 days after onset. Twenty-five days after onset, he began to develop a fever, with a maximum temperature of more than 39 degrees. His hemoglobin and platelet counts were normal on admission but gradually decreased as low as 67 g/l and 10 × 10^9^/l, respectively, after admission. Pathogen detection tests, including blood culture and the atypical pathogens and G/GM test, were negative. Hepatitis B virus (HBV) and hepatitis C virus (HCV) were negative. HIV antibody and HHV-8 PCR were negative. The polyclonal light chains in both the blood and urine were increased. The levels of serum kappa and lambda light chain were 170 mg/l (3.30–19.40) and 208.25 mg/l (5.71–26.3), respectively (kappa/lambda: 0.82). However, the levels of IgG and IgA were within the normal range, and the levels of IgM and complement 3/4 were below normal. Antibodies, including ANA, RF, ANCA, and platelet-associated IgG, were undetectable. His serum VEGF and IL-6 levels were > 800 pg/ml (0.0–142.0) and 11.5 ng/ml (0.0–7.0), respectively. An abdominal puncture was performed, and 1000 ml of ascites fluid was drained 40 days after onset. The total number of cells in ascites was 121 and included 40 nucleated cells (34 mononuclear cells and 6 multinuclear cells). Total protein, albumin and glucose levels in ascites were 28.0 g, 14.4 g/l and 14.96 mmol/l, respectively. The IL-6 level in ascites reached 1022 pg/ml (0.0–7.0). Bone marrow biopsy showed marked hyperplasia of granulocytes and megakaryocytes, and reticular fiber staining was negative. PET/CT showed that FDG activity was increased in the bilateral neck, bilateral axillary areas, mediastinum, bilateral hilar lymph nodes, bilateral parailiac artery, and bilateral inguinal lymph nodes and did not reveal any carcinoma. Pathology of the lymph node puncture showed plasmacytosis. After ineffective antibiotic treatment with meropenem, he was given intravenous methylprednisolone (60 mg qd) 36 days after onset combined with intravenous immunoglobulin (20 g qd for 5 days). At the early stage of glucocorticoid therapy, his pleural effusion and ascites decreased significantly, accompanied by a general improvement. However, 46 days after onset, his temperature increased again, with a mild increase in bilirubin and a significant decrease in blood cells. Therefore, lymphadenectomy was performed, and histopathology showed a massive hemorrhage in the lymph nodes with a normal lymph node structure, decreased lymphoid follicles, an atrophied germinal center, a concentric circle arrangement in the partial mantle zone, the proliferation of small vessels, enlargement of the paracortical zone, and flaky or focal infiltration of mature plasma cells. These findings are consistent with the mixed histopathological subtype of iMCD. After a multidisciplinary consultation, the patient was diagnosed with iMCD and was believed to have hemophagocytic syndrome according to the decreased NK cell activity (14.09%) and increased soluble CD25 level (10,615 pg/ml). For further care, he was referred to our hospital which was famous for being specialized in hematology. Based on his clinical manifestations (fever, ascites, thrombocytopenia, lymphadenopathy and progressive renal insufficiency) and lymph node biopsy (atrophied germinal center and the proliferation of small vessels), we diagnosed the patient with TAFRO syndrome. As the CMV DNA contents of the serum and lymphocytes were increased (4.76 × 10^4^ cp/ml and 4.25 × 10^6^ cp/ml, respectively) 53 days after onset, ganciclovir was added to his treatment plan. With an improvement in renal function, hemodiafiltration was stopped 2 months after onset. However, proteinuria was still serious, with 24-h urine protein reaching 31 g, so renal biopsy was performed 3 months after onset. Histopathology revealed 11 glomeruli in which a membranoproliferative glomerulonephritis (MPGN) pattern was observed, with diffuse endocapillary proliferation, mesangial proliferation, fuchsinophilic protein deposition in the subendothelial and mesangial areas by Masson stain, double contours and microthrombosis seen in some capillary loops, vacuole and granular degeneration of renal tubular epithelial cells, and arterial endothelial cell proliferation with thickened walls and a narrowed lumen. Immunofluorescence showed two glomeruli with immune complex deposition (IgA ++, IgG +++, IgM ++, C1q ++, C3 +++, FRA -, Kappa ++, Lambda ++), which were deposited with clumps and granules along the mesangial zero and capillary wall. The IgG subtype detection of immune complexes showed IgG1 ++, IgG2 ++, IgG3 and IgG4 -. Electron microscopy revealed intramembranous, subendothelial and partially subepithelial electron-dense deposits and diffuse podocyte foot processes (Fig. 4). His renal histopathology were consistent with those of cases with TAFRO syndrome reported in literatures, which further confirmed his diagnosis of TAFRO syndrome. One hundred twenty days after onset, therapy with intravenous rituximab (CD20 B-cell monoclonal antibody, from Roche) was initiated (375 mg/m^2^ once a week for the first month and then once evert 2 months). The patient has not been hospitalized since the fifth course of treatment. At the latest follow-up, his hemoglobin level was 133 g/L, his platelet level was 309 × 10^9^/L, his creatinine level was 103 μmol/L, and his body temperature and urine volume were normal (Fig. 5).

## Discussion and conclusion

These two patients were treated in our hospital consecutively, and case 1 was admitted approximately 1 month after case 2. Because of the lack of understanding of this disease entity in clinicians, the diagnostic process of case 2 was more tortuous than that of case 1. When he was referred to our department, he visited a hematology specialist clinic in our hospital, and TAFRO syndrome was suspected. When case 1 was admitted to our hospital, we did not establish a connection between the two for a long time because of their distinct clinical manifestations.

Although the onset and course of these two patients were very different, the common clinical manifestations could be summarized as fever, polyserositis, anemia, thrombocytopenia, lymphadenopathy of multiple nodes and progressive renal insufficiency. The histopathology of lymph nodes in both patients showed atrophy of the germinal center and hyperplasia of small vessels. Fortunately, renal biopsies were performed in both patients and advanced our understanding of their clinical manifestations. These two cases reminded us that it is very important to actively create conditions to seek evidence when we are unable to provide a reasonable explanation for a patient’s symptoms or do not know enough about his/her disease.

Clinicians usually encounter many challenges in diagnosing iMCD posed by nonspecific symptoms and an unclear etiology. In contrast to TAFRO-iMCD, TAFRO-iMCD may be readily recognized through its easily identifiable clinical symptoms and pathological features. Lymph node histology in patients with TAFRO syndrome is usually diagnosed as the HV or mixed-type, but cases of iMCD-NOS usually show classic PC-type features. In addition, polyclonal hypergammaglobulinemia, lymphadenopathy of multiple nodes, thrombocytosis, and a chronic clinical course are characteristics of iMCD-NOS, whereas a normal immunoglobulin level, thrombocytopenia, relatively small lymphadenopathy, marked polyserositis and edema, and acute or subacute onset and clinical course are characteristics of TAFRO syndrome [16]. In the report of Iwaki et al., the serum IL-6 level was lower for TAFRO-iMCD than for iMCD-NOS; however, the plasma level of VEGF was significantly increased for TAFRO-iMCD [15]. This result was also confirmed in our unpublished analysis of iMCD.

In CD, renal complications seem to be uncommon. In the study described by El Karoui K et al., 9 of 15 patients with HIV-negative CD had small-vessel lesions (60%) with endothelial swelling, glomerular capillary-loop double contours and mesangiolysis, followed by AA amyloidosis (3/15, 20%) [17]. Xu et al. analyzed the data of 76 HIV-negative CD patients, 19 of whom were complicated with renal involvement, including hematuria (14/19,74%), proteinuria (18/19,95%), and acute (12/19,63%) or chronic (1/19,5%) renal failure. Eleven of the 19 patients received a renal biopsy examination, and the pathological findings included TMA-like lesions in six patients (55%), crescentic glomerulonephritis in three patients (27%), minimal change disease in one patient (9%), chronic tubulointerstitial nephropathy in one patient (9%) and membranous nephropathy in one patient (9%, combined with chronic tubulointerstitial nephropathy). Three of the 11 patients had thrombocytopenia, whose histopathological types of lymph node biopsy were all mixed variants, and renal pathological results included TMA-like lesions (2 patients) and chronic tubulointerstitial nephropathy (1 patient) [18].

Renal dysfunction is very common in patients with TAFRO syndrome, with the two main patterns of renal pathological changes being described in recent literature as TMA-like glomerulopathy and MPGN [19–28]. On light microscopy, MPGN and chronic TMA share similar histological patterns: mesangial proliferation, double contours and endocapillary proliferation. The detection of intense immune deposits on immunofluorescence and/or electron-dense deposits favors a diagnosis of MPGN [29, 30]. The descriptions of renal biopsies of patients with TAFRO syndrome are similar to those of small-vessel lesions reported previously in iMCD [17].

Disease onset and course were very different in the two cases reported herein. Case 2 showed massive ascites, severe anasarca, and renal involvement characterized by nephrotic syndrome and massive proteinuria. While the edema in case 1 was mild, oliguria was not obvious, so renal involvement did not receive attention before admission until the laboratory examination showed severe renal failure. The clinical manifestations of the patients were consistent with the results of lymph node biopsy and renal biopsy. Renal biopsy revealed nearly half of glomeruli exhibiting ischemic sclerosis in case 1, while mesangial and endothelial proliferation was much slighter than that in case 2. The infiltration of mature plasma cells in case 1 was less than that in case 2. It is worth noting that in case 2, although the level of IL-6 in the serum was slightly increased, the level of IL-6 in ascites was significantly increased; therefore, it is of significance for us to further explore the pathogenesis of ascites.

Regarding the prognosis of patients with TAFRO syndrome, additional cases still need to be reported. Tanaka et al. described 10 patients with TAFRO syndrome who required hemodialysis [23]. Both proteinuria and hematuria tended to be mild; nephrotic-range proteinuria was reported only in 1 patient, and only 1 patient died over the follow-up. In the literature review by Leurs A et al., outcomes tended to be worse when hemodialysis was necessary (44% of deaths compared to 5% when hemodialysis was not needed), without differences in outcomes between cases described as MPGN and TMA-like [31]. After hormone and monoclonal antibody treatment, the conditions of the two patients improved significantly, but they still did not achieve complete remission and needed further treatment and follow-up.

In conclusion, as a rare disease, the diagnosis of TAFRO syndrome is based mainly on clinical manifestations and lymph node biopsies. A reliable early diagnosis and appropriate rapid treatment are essential to improve the outcomes of patients. Clinicians should deepen their understanding of this disease and similar conditions. Once the disease is suspected, lymph node biopsies should be performed as soon as possible. In addition, renal biopsies should be actively performed in patients with renal involvement.

## Data Availability

The datasets used and/or analyzed during the current study are available from the corresponding author on reasonable request.

## Abbreviations

CD: Castleman disease
MCD: Multicentric CD
iMCD: Idiopathic multicentric CD
TAFRO: Thrombocytopenia, anasarca, fever, reticulin myelofibrosis and organomegaly
iMCD-NOS: Idiopathic multicentric Castleman disease-not otherwise specified
HV: Hyaline vascular
IL: Interleukin
VEGF: Vascular endothelial growth factor
TMA: Thrombotic microangiopathy
TCZ: Tocilizumab
MPGN: Membranoproliferative glomerulonephritis
